# The Provision of Care for Patients with Tuberculosis and Diabetes Mellitus Multi-Morbidity in Addis Ababa, Ethiopia: An Analysis of Patients’ Perspectives

**DOI:** 10.3390/ijerph23070925

**Published:** 2026-07-18

**Authors:** Sisay Tiroro Salato, Keitshepile Geoffrey Setswe

**Affiliations:** 1 Ethiopia Regional Learning Center, University of South Africa, Addis Ababa 1165, Ethiopia; 64053989@mylife.unisa.ac.za; 2 Department of Health Studies, University of South Africa, Pretoria 0002, South Africa; 3 Bafokeng Health & Demographic Surveillance (BAMMISHO) Node, The Aurum Institute, Johannesburg 2001, South Africa

**Keywords:** care, diabetes mellitus, tuberculosis, multi-morbidity, health services, Addis Ababa

## Abstract

**Highlights:**

**Public health relevance—How does this work relate to a public health issue?**

- Integrated care transforms care systems to promote person-centred care for patients with TB-DM co-morbidity.
- It improves patients’ experiences of care received and outcomes at health facilities.

**Public health significance—Why is this work of significance to public health?**

- Integrated care improves the coordination of care and ensures streamlined care pathways for patients with TB-DM.
- It reduces unnecessary use of institutional care and costs.

**Public health implications—What are the key implications or messages for practitioners, policy makers and/or researchers in public health?**

- Integrated care supports workforce development to improve the quality of care provided to patients with TB-DM.
- It addresses inequalities in care received by patients with either TB, DM or both.

**Abstract:**

Providing care for effective management of tuberculosis (TB) and diabetes mellitus (DM) is a challenge, particularly in resource-limited settings. This study assessed factors affecting the provision of care for patients with TB-DM in Addis Ababa, Ethiopia, using the six elements of the SELFIE—**S**ustainable int**E**grated chronic care mode**L**s for **F**inanc**I**ng and performanc**E** framework. A health facility-based cross-sectional study was conducted with randomly selected patients with TB-DM multi-morbidity. Data were analysed using the Statistical Package for Social Sciences (SPSS) version 27. Logistic regression was employed to identify factors influencing TB-DM care provision. A total of 357 respondents participated, with a response rate of 96.5%. The mean age of the respondents was 49.8 years. Only 13.4% of patients received good TB-DM services. Key factors influencing TB-DM care were (a) insufficient counseling on the proper use of medication (AOR = 2.6, CI: 1.1–6.6, *p* = 0.035) and the risk of TB for DM patients (AOR = 10, CI: 3.7–27, *p* < 0.001), (b) leadership and governance including supportive leadership for TB-DM care (77.1%), organized TB-DM care (62.5%), the presence of a care policy (77.1%), and continuity of care (58.3%), (c) workforce factors such as the presence of a multidisciplinary team (60.4%), TB-DM service coordinator (58.3%), HCWs with adequate knowledge (87.5%), (d) costs of health services are fair (75%) and (e) lack of technologies such as EMR (88%) and lack of medical products to treat TB-DM (64.7%) delays care for TB-DM. Most TB-DM patients in Addis Ababa had limited access to care. The continuous monitoring of services can facilitate the identification of care gaps, thereby guiding the implementation of improved interventions.

## 1. Background

The World Health Organization (WHO) reported over 10.6 million new cases of tuberculosis (TB) worldwide in 2021, resulting in 1.4 million deaths. Ethiopia, one of the world’s high-burden countries, has an incidence rate of 126 per 100,000 people [1]. In 2020, approximately 530 million adults worldwide were living with diabetes, with over a quarter living in low- and middle-income countries (LMICs); approximately 1.4 million adults in Ethiopia are living with diabetes [2].

Studies have confirmed the epidemiological association between TB and DM [3,4,5]. The coexistence of TB and type 2 diabetes mellitus (T2DM) represents a significant public health challenge in developing countries [6,7,8]. This dual burden represents a significant public health concern, as it has the potential to exacerbate health outcomes, complicate treatment strategies [9,10], and increase the risk of treatment failure and mortality [11,12]. In 2021, the WHO reported that DM was a contributing factor in more than 2.72% of TB cases in Ethiopia [1]. In addition, a systematic review and cross-sectional study conducted in Ethiopia revealed that 12.7% and 15.8% of cases had concurrent TB and DM, respectively [8,13]. The Ethiopian National Strategic Plan for Tuberculosis, set the objective of furnishing client-centered integrated TB and DM services, because the proportion of participants who receive TB and DM care in the country is low [14].

Healthcare services are designed to provide coordinated care that addresses multiple health needs simultaneously, which is essential for the effective management of multi-morbidity [15,16]. Multi-morbidity is the co-occurrence of two or more chronic conditions in an individual [17,18]. In the context of Ethiopia, the health system faces significant challenges in providing healthcare services, mainly due to resource constraints, inadequate infrastructure and a lack of trained health workers [19]. Despite these challenges, the integration of TB and DM care is imperative for improving patient outcomes and ensuring comprehensive management of both diseases [20,21].

Patients frequently encounter a multitude of obstacles when attempting to access healthcare services. These impediments include geographic isolation, financial constraints, and systematic inefficiencies within the healthcare system [22,23]. Moreover, deficiencies in the quality of care, such as inconsistencies in treatment protocols and inadequate follow-up, impede the effective management of patients with TB and DM [16,19,22]. Understanding patients’ perspectives is crucial to TB-DM service delivery, as the literature has shown that experiences and attitudes have a significant impact on care seeking and adherence, whereas previous studies have emphasized the importance of incorporating patient perspectives in the development of ideal healthcare policies and practices [24,25,26].

Cultural beliefs pertaining to TB and DM may influence patients’ health-seeking behaviors [27]. In particular, the stigmatization of TB can act as a deterrent to individuals seeking necessary healthcare services. Moreover, research has demonstrated that the socio-economic, income level, educational, and employment characteristics of patients can influence their ability to access healthcare services, which in turn can affect their overall health outcomes [28]. Despite the existing body of literature, there is a paucity of research investigating health system factors affecting the delivery of health services for patients with TB and T2DM multi-morbidity from the perspective of patients in Addis Ababa, Ethiopia.

This study was aimed at assessing factors affecting the provision of health services for patients with the multi-morbidity of TB-DM using the six elements of the SELFIE—**S**ustainable int**E**grated chronic care mode**L**s for **F**inanc**I**ng and performanc**E** framework [29].

## 2. Materials and Methods

### 2.1. Study Setting

The study was conducted in Addis Ababa, the capital of Ethiopia, with a population exceeding 3.8 million people. Administratively, the city is divided into ten sub-cities and 114 districts. The city has a total of 103 registered public health facilities, comprising 11 hospitals and 92 health centers [30], which provide general services to the population.

### 2.2. Study Population

According to data from the Addis Ababa City Administration Health Bureau (Ethiopia Ministry of Health, 2015) [31], there were 2563 adult patients with pulmonary TB and 405 who had been diagnosed with DM who actively sought health services at selected public health facilities in the city during the study period.

### 2.3. Study Design

A cross-sectional study was conducted with patients receiving care for TB and DM at health facilities in Addis Ababa using the six elements of the SELFIE (Sustainable intEgrated chronic care modeLs for FinancIng and performancE) framework.

### 2.4. Sampling Design and Sample Size Estimation

A three-stage cluster sampling design was used to select a sample.

In stage 1, 6 hospitals and 46 health centres were proportionally selected as a 50% sample from 11 hospitals and 92 health centres in Addis Ababa.

In stage 2, the baseline/source population for this study was 2563 patients with PTB and 405 with DM in the six hospitals and 46 health centres in the study community.

In stage 3, the sample size was calculated using a single population proportion formula at a 95% confidence interval and a 5% margin of error, and due to the absence of prior studies on the integration of TB-T2DM services, 50% was assumed to obtain maximum sample size.

$$n=\frac{Z\alpha {/}_{2}\times P(1-P)}{d}$$

$$n=\frac{(1.96)2\times 0.5\times 0.5}{(0.05)2}$$

where

Zα/_2_ = the value from standard normal distribution table for Z, which is 1.96,
P = the proportion of TB-T2DM service integration (50%),
1 − P = proportion of TB-T2DM service non-integration (50%), and
d = the margin of error

The total estimated sample size was 384 patients with TB-DM multimorbidity. Based on this sample size, we calculated the number of participants to be selected from each health facility.

### 2.5. Inclusion and Exclusion Criteria

The study included adult patients with pulmonary TB-T2DM but excluded those who declined to provide consent, were too critically ill at the time, exhibited clinical signs of SARS-CoV-2 infection, or had extra-pulmonary forms of TB-T2DM.

#### Operational Definition

TB-DM care was defined as the provision of coordinated services for both conditions within the same facility, including joint consultation, shared treatment planning and/or synchronized follow-up.

### 2.6. Data Collection Procedures

The survey questionnaire was adapted from the SELFIE framework which contained the following key factors: (a) service delivery—focusing on the delivery of care services to individuals with TB-DM multi-morbidity, (b) leadership & governance addressing the leadership and governance structures that support integrated care, (c) workforce involving HCWs involved in providing care and support to individuals with multi-morbidity, (d) financing of integrated care services, (e) technologies & medical products to support integrated care and (f) information and research to support the development and implementation of integrated care for TB-DM patients [29].

The questionnaire was piloted on an internal sample of 20 TB-DM clients to identify questions that do not make sense to participants, or problems with the questionnaire that might lead to biased answers. Trained data collectors conducted face-to-face interviews with selected patients at sampled health facilities, during the participants’ scheduled visit for TB-DM care. The study was conducted between June and December 2023.

### 2.7. Data Management and Analysis

The collected data were checked, edited, coded and entered into the Statistical Package for Social Sciences (SPSS) version 32, for analysis. Descriptive analyses, including the calculations of means, medians, standard deviations, and frequency distribution tables, were conducted. Logistic regression analysis was conducted to identify the factors affecting the provision of health care for patients with TB and DM. The strength of the association was quantified via the adjusted odds ratio and a *p* value less than 0.05 was considered statistically significant.

### 2.8. Ethical Considerations

The College Research Ethics Committee (CREC) of the University of South Africa (Reference Number: 240815-052) and the Institutional Review Board of the Addis Ababa Health Bureau (Reference Number: A/A9869/227) granted ethical approval for this research. All the methods were carried out in accordance with the relevant guidelines and regulations. In addition, all the health facilities provided written permission.

## 3. Results

### 3.1. Demographic Characteristics of Participants

A total of 357 patients were enrolled in the study, with a response rate of 93%. The age of the participants ranged from 23–87 years, with a mean age of 49.87 ± 14.046 years. One-third of the participants were 60 years of age or older, while more than a quarter were between the ages of 40 and 49. Most of the participants were male (54.3%) and resided in urban areas (94.7%). The educational background of the participants was diverse, with 40.1% having obtained a diploma or higher education, 27.2% having completed secondary education, 20.4% having completed primary education, and 12.3% having no formal education (Table 1).

### 3.2. Patient Perspectives on Service Delivery Factors Affecting Care for TB-DM

**Figure 1:**
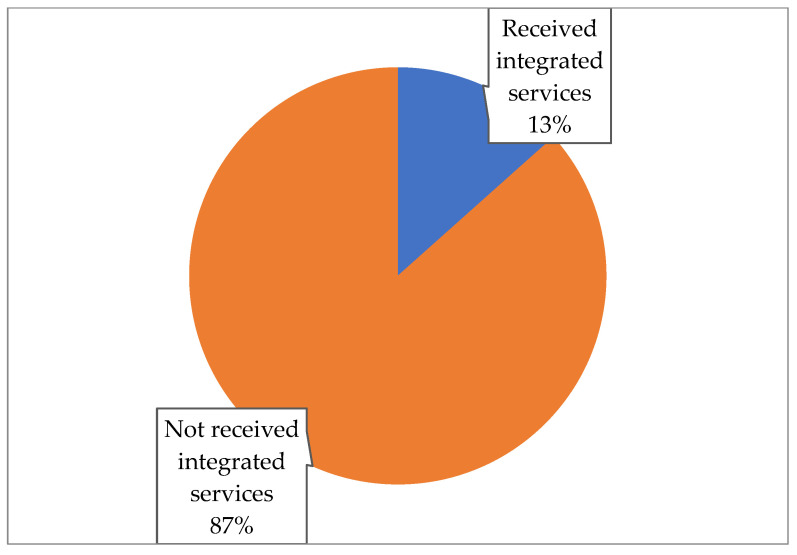
TB-DM patients with multi-morbidity who received healthcare services.

In this study, health services for TB and DM were delivered to only 13.4% (95% CI of 10.1% to 17.4%) of the patients across the 25% of the surveyed health facilities (in three hospitals and eleven health centers) (Figure 1).

The results of the multivariate logistic regression analysis indicated that the provision of counseling on the proper use of medications (AOR = 2.6, CI: 1.1–6.6, *p* < 0.001) and counseling about the risk of TB infection for diabetes patients (AOR = 10, CI: 3.7–27, *p* < 0.001) influenced the provision of TB and DM services (Table 2).

### 3.3. Leadership and Governance Factors Influencing the Provision of Care for Patients with TB-DM

The presence of supportive leadership and governance was found to have influence in receiving TB-DM healthcare services. Over three-quarters (77.1%) of TB-DM patients said they had supportive leadership in their health facilities, 62.5% of patients had received organised TB-DM care, 77.1% of respondents reported the existence of a policy for TB-DM care, while 58.3% of respondents said they had received continuous care for TB-DM (Table 3).

### 3.4. Workforce Factors Affecting Care for Patients with TB-DM

Table 4 indicates that several health workforce factors positively influence health service provision for patients with TB-DM. Approximately 60.4% of patients said their care is improved if facilities have a multidisciplinary team, 58.3% if the facilities have a TB-DM service coordinator, 87.5% if health workers have adequate knowledge, and 50% if facilities involved informal caregivers in TB-DM.

### 3.5. Financial Factors Affecting Care for Patients with TB-DM

In Ethiopia, the costs of health services are covered by a Community-Based Health Insurance (CBHI) program which covers 80% of citizens. In 75% of facilities which have no mechanisms for reimbursing their healthcare costs and in 75% of health facilities where healthcare services are fair patients said they were more likely to receive decent healthcare for TB-DM (Table 5).

### 3.6. Technologies & Medical Products Used for the Provision of Care for Patients with TB-DM

Only 12% of respondents said health facilities were using electronic medical record system, while 24.1% reported that their health facilities had access to technologies such as glucometers for monitoring blood sugar levels, acid-fast bacilli (AFB) tests to detect TB, GeneXpert to diagnose TB and drug resistance, chest X-rays for diagnosing TB, while 75.9% of patients did not have access to these technologies.

Around 65% of patients reported that their health facility did not have access to medical products for treating TB-DM, while 35.3% reported that their health facility had access to these products. These drugs are essential for the effective treatment and management of TB-DM.

Only 14.8% patients reported that their health facility had mechanisms to monitor the burden and care of TB-DM while a significant majority (85.2%) of patients reported that their health facilities did not have these mechanisms in place (Table 6).

### 3.7. How Information and Research Are Used for the Provision of Care for Patients with TB-DM

Approximately 42.0% of TB-DM patients had access to individualised data for their condition, while 58.0% of patients did not have access to this data. More than a third of respondents, 35.6%, said that the individualised data they had helped to predict their level of risk to TB-DM.

Less than two-thirds, 61.3% of respondents, reported that health facilities had systems in place to protect the privacy of their data, while 38.7% did not have such systems in place. Over three-quarters of respondents, 78.4%, felt that access to their health information was important (Table 7).

## 4. Discussion

This study investigated factors affecting the provision of healthcare services for patients with TB-DM in Addis Ababa, Ethiopia, using the six elements of the SELFIE framework, providing a system-level perspective from patients’ experience.

### 4.1. Service Delivery Factors

Despite the Ethiopian National Strategic Plan for Tuberculosis, which set the objective of furnishing client-centered integrated TB and DM services, the findings of this study indicate that a mere 13.4% of participants received TB and DM care in Addis Ababa. This can be attributed to a lack of DM services at the health facility level, a lack of trained personnel, and a lack of essential resources for the care of patients with DM [32,33]. Therefore, these findings suggest that only a limited number of facilities are adequately equipped to provide both TB and DM services effectively. A comparable study conducted in India revealed that over half of the patients with TB and DM multi-morbidity received health care [34], which is a greater proportion than that reported in the current study. This discrepancy in provision rates may be due to differences in health system policies in different regions, as these policies play a critical role in determining the level of collaboration between TB and DM health services.

### 4.2. Workforce Factors

The study revealed that the provision of counseling and the appropriate use of medications influence TB-DM care. Patients diagnosed with both TB and DM who received counseling on the proper use of their medications perceived receiving good care. Evidence from empirical studies indicates that the implementation of effective health education and counseling strategies influences adherence to treatment regimens, more effective management of comorbidities, and overall improved patient outcomes [35]. It is plausible that this counseling provides patients with necessary information regarding their conditions and medications, which could ultimately result in enhanced adherence to treatment regimens.

### 4.3. Leadership and Organisation

The findings of this survey indicate that the availability of organized care for individuals with TB and DM influences the provision of care for patients with both conditions. The provision of TB and DM services is important, particularly in LMICs, where both diseases have a profound impact on public health. A study from Zimbabwe demonstrated a 57% increase in DM screening among TB patients when services were organised in comparison with less organised settings [36].

Despite the benefits, a study conducted in LMIC has indicated that the actual integration of services remains minimal. A review revealed that limited awareness of TB-DM multi-morbidity represents a significant obstacle to effective integration [37]. This underlines the necessity for targeted interventions to improve awareness and training among healthcare providers. Furthermore, this finding is supported by the WHO collaborative framework for the care and control of TB and DM, which underscore the need for harmonized efforts across health systems to address both conditions effectively [38]. The implementation of this framework has demonstrated the feasibility and efficacy of bi-directional screening and management, particularly in rural settings where resources may be constrained [39,40].

The results of this study indicate that a policy on effective care for patients with TB and DM influences the provision of coordinated services. This finding is consistent with the conclusions of several other studies that emphasize the importance of policies in enabling effective healthcare provision. The International Union Against Tuberculosis and Lung Disease (The Union) and the WHO framework for the management of TB and DM underscores the need for coordinated responses to both diseases. A systematic review of the literature revealed that countries implementing coordinated responses reported the implementation of feasible and effective bi-directional interventions for the management of TB and DM [41].

The availability of a supportive policy framework was a critical factor in establishing collaboration between TB and DM control programs, which ultimately resulted in improved service delivery [42]. In Ghana, the implementation of a supportive policy by the National Tuberculosis Control Program (NTP) facilitated the provision of good TB and DM care. The implementation of bidirectional screening was made possible by the appointment of a TB task-shifting officer, whose responsibilities included training and coordinating screening efforts across various healthcare settings, including diabetes clinics [42]. This initiative demonstrates how a well-defined policy can facilitate operational modifications that enhance care provision. The evidence base is still evolving, particularly with regard to the cost-effectiveness and operational challenges influencing the implementation of TB-DM care models [43,44].

The provision of a continuum of care is important for the effective management of TB and DM multi-morbidity. Moreover, a pilot study conducted in India demonstrated the feasibility and efficacy of a program that combined the management of TB and DM within the context of primary healthcare [45]. Despite the benefits, barriers persist in ensuring a seamless continuum of care. A recent study indicated that a significant proportion of patients with TB are not screened for DM. In some settings, only 15% of patients have undergone adequate screening for DM [46]. This deficiency in screening can result in delayed diagnoses and inadequate management, thereby underscoring the necessity for policies that endorse routine screening as an integral component of TB treatment protocols.

### 4.4. Financing

Findings in this study suggest that the existence of reimbursement mechanisms and ensuring the fairness of healthcare costs play a crucial role in promoting healthcare provision for TB-DM. These findings are consistent with a study conducted in the Philippines [47], which also highlighted the importance of reimbursement mechanisms and fairness of healthcare costs in achieving better healthcare services. Overall, these findings highlight the importance of addressing health financing systems, including reimbursement mechanisms and equity in health care costs, to improve the provision of health services for TB-diabetes mellitus patients.

### 4.5. Technologies & Medical Products

The results showed that health facilities using an electronic medical record (EMR) system positively influence care for patients with TB-DM. Similarly, facilities that had access to TB and DM diagnostic technologies, such as GeneXpert and glucometers, influence TB-DM clients. This finding is consistent with a previous study in Tanzania [48], which demonstrated the critical role of technologies such as GeneXpert and glucometers in influencing the quality of TB and DM services.

### 4.6. Health Information and Research

Overall, the findings in this study underscore the need for effective data management, privacy safeguards and patient engagement in TB-DM care. The importance of access to TB-DM information for patients influences healthcare provision. When patients are well informed about their condition and have access to relevant information, they can be active participants in their own care, make informed decisions and collaborate effectively with healthcare providers. This finding is consistent with a study conducted in South Africa [49].

#### Strengths and Limitations of the Study

One of the key strengths of this study was the use of the validated SELFIE framework to evaluate the health system factors influencing integrated TB-DM care. To the best of our knowledge, this was one of the few studies to undertake a systematic investigation of this important but often overlooked research area in many developing countries.

Importantly, the present study was conducted at urban public health facilities, which may limit the generalizability of the findings to rural contexts or private health services. As a quantitative study employing predefined responses, this approach may prove inadequate for capturing the subtleties of patient experiences and perspectives. A focus on standardized measures alone carries the risk of oversimplification, as it fails to account for the impacts of individuality, culture, and emotion on health.

## 5. Conclusions

The study findings indicated that only a minority of patients in Addis Ababa, Ethiopia, had access to TB-DM services. From the patients’ perspective, the identified factors affecting the provision of services included the lack of provision of counseling on the proper use of medications, lack of counseling on the risk of TB infections for DM patients, the absence of organized TB-DM services, and the lack of integrated policies and the absence of a continuum of care.

To enhance care for patients with TB and DM in Addis Ababa, it is imperative that policymakers prioritize the strategies addressing TB and DM. The continuous monitoring of services and periodic assessments can facilitate the identification of care gaps, thereby guiding the implementation of improved interventions. Future studies should employ a mixed qualitative and quantitative methodology across multiple rural private healthcare sites, which would allow for a more in-depth exploration of the patient’s perspective.

Again, the study shows the many deficiencies of TBDM care in Addis Ababa. Lack of integration is one among many others.

## Figures and Tables

**Table 1:** Socio-demographic characteristics of the study participants (n = 357).

Variables	Frequency	Percentage (%)
Age		
18–29	23	6.4
30–39	76	21.3
40–49	91	25.5
50–59	61	17.1
≥60	194	54.3
Gender		
Male	194	54.3
Female	163	45.7
Residence		
Urban	338	94.7
Rural	19	5.3
Educational status		
No schooling	44	12.3
Primary school	73	20.4
Secondary school	97	27.2
Diploma and above	140	40.1
Occupational status		
Employed	190	53.2
Unemployed	37	10.4
Housewife	49	13.7
Retired	59	16.5
Other	22	6.2
Household income in Ethiopian Birr		
≤2500	75	24.8
2501–4500	51	16.9
4501–5500	18	6.0
≥5501	158	52.3

**Table 2:** Health service delivery factors affecting the provision of TB-DM services in Addis Ababa, Ethiopia, 2023.

Service Delivery Components	Health Care Service		OR 95%CI	*p* Value	AOR 95%CI	*p* Value
	Yes	No				
Educational status	48(13.5%)	309 (86.5%)				
No schooling	9	35	0.32 (0.12–0.83)	0.021	0.5 (0.11–2.6)	0.42
Primary school	12	61	0.42 (0.17–1.01)	0.054	0.6 (0.14–2.3)	0.43
Secondary school	16	81	0.42 (0.18–0.95)	0.038	0.69 (0.21–2)	0.52
Diploma and above	11	132	1		1	
Household monthly income						
<2500	15	60	0.36 (0.16–0.8)	0.012	0.9 (0.27–3.3)	0.94
2501–4500	10	41	0.37 (0.15–0.9)	0.028	0.79(0.2–3)	0.73
4501–5500	5	13	0.23 (0.07–0.75)	0.015	0.2 (0.04–0.9)	0.046
≥5501	13	145	1		1	
Provision of counseling on proper use of medications						
Yes	33	128	1.0			
No	15	181	3.11 (1.6–5.9)	<0.001	2.6 (1.1–6.6)	0.035
Provision of counseling about risk of TB infection						
Yes	27	26	1.0			
No	21	283	13.99 (6.9–28)	<0.001	10 (3.7–27)	<0.001

**Table 3:** Leadership and governance factors influencing the provision of health care for TB-DM patients in Addis Ababa, Ethiopia, 2023.

Leadership & Governance	Health Service Provision		OR	95%CI	*p*-Value
	Yes	No			
There is supportive leadership for the care of TB-DM patients					
Yes	37 (77.1%)	88 (28.5%)	1.0		
No	11 (22.9%)	221 (71.5%)	8.45	4.12–17.3	<0.001
There is organized TB-DM services					
Yes	30 (62.5%)	64 (20.7%)	1.0		
No	18 (37.5%)	245 (79.3%)	6.38	3.3–12.1	<0.001
There is a policy to provide care for TB-DM					
Yes	37 (77.1%)	76 (24.6%)	1.0		
No	11 (22.9%)	233 (75.4%)	10.31	5–21.2	<0.001
There is continuous care for TB-DM					
Yes	28 (58.3%)	68 (22.0%)	1.0		
No	20 (41.7%)	241 (78.0%)	4.9	2.6–9.3	<0.001

**Table 4:** How health workforce influences the provision of care for patients with TB-DM in Addis Ababa, Ethiopia, 2023.

Health Workforce	Healthcare Service		OR	95%CI	*p*-Value
	Yes	No			
There is a multidisciplinary team.					
Yes	29 (60.4%)	21 (6.8%)	1.0		
No	19 (39.6%)	288 (93.2%)	20.93	10.1–43.4	<0.001
There is a TB-DM service coordinator.					
Yes	28 (58.3%)	35 (11.3%)	1.0		
No	20 (41.7%)	274 (88.7%)	10.96	5.6–21.5	<0.001
Health workers have adequate knowledge					
Yes	42 (87.5%)	165 (53.4%)	1.0		
No	6 (12.5%)	144 (46.6%)	6.1	2.5–14.8	<0.001
There is involvement of informal care givers					
Yes	24 (50.0%)	22 (7.1%)	1.0		
No	24 (50.0%)	287 (92.9%)	13	6.4–26.6	<0.001

**Table 5:** Health financing factors influencing the provision of TB-DM care in Addis Ababa, Ethiopia 2023.

Health Financing	Healthcare Service		OR	95%CI	*p*-Value
	Yes (n, %)	No (n, %)			
Health facilities will reimburse the costs of the services.					
Yes	12 (25.0%)	21 (6.8%)	1.0		
No	36 (75.0%)	288 (93.2%)	4.6	2.07–10.06	<0.001
Costs of the health services are fair					
Yes	36 (75.0%)	110 (35.6%)	1.0		
No	12 (25.0%)	199 (64.4%)	5.42	2.7–10.86	<0.001

**Table 6:** Access to technologies and medical products among patients with TB-DM in Addis Ababa, Ethiopia, 2023.

Health Technologies and Medical Products	Frequency	Percentage
Health facilities are using electronic medical record system		
Yes	43	12.0%
No	314	88.0%
Facilities use technologies to diagnose TB and DM		
Yes	86	24.1%
No	271	75.9%
There are mechanisms to monitor care for patients with TB-DM		
Yes	53	14.8%
No	304	85.2%
Health facilities have access to medical products to treat TB-DM		
Yes	126	35.3%
No	231	64.7%

**Table 7:** Access to health information and research among patients with TB-DM in Addis Ababa, Ethiopia, 2023.

Health Information and Research	Yes	Percentage
TB-DM patients have individualized data for TB-DM		
Yes	150	42.0%
No	207	58.0%
There are mechanisms to predict individual risk		
Yes	127	35.6%
No	230	64.4%
Health facilities have a system to protect privacy of your data		
Yes	219	61.3%
No	138	38.7%
Access to information is important for patients with TB-DM		
Yes	280	78.4%
No	77	21.6%

## Data Availability

The datasets generated and analysed during this study are not publicly available due to confidentiality agreements. However, anonymised data, questionnaires, and analysis codes are available from the corresponding author upon reasonable request for replication or secondary analysis.

## Abbreviations

The following abbreviations are used in this manuscript:

AOR	Adjusted Odds Ratio
CI	Confidence Interval
DM	Diabetes Mellitus
DOTS	Directly Observed Treatment Short course
LMICs	Low- and Middle-Income Countries
SARS-CoV-2	Sever Acute Respiratory Syndrome Coronavirus 2
SELFIE	Sustainable intEgrated chronic care modeLs for multi-morbidity: delivery, FinancIng and performancE
TB	Tuberculosis
T2DM	Type 2 Diabetes Mellitus
TBDM	Tuberculosis- Diabetes Mellitus
WHO	World Health Organisation
